# Supplementary material for: Comparative effects of French Contrast Method vs. Complex Training on explosive power and its endurance in youth badminton athletes
Source: PLoS One. 2025 Dec 5;20(12):e0338383. doi: 10.1371/journal.pone.0338383 (PMC12680223; doi:10.1371/journal.pone.0338383)
Supplement: Attachment — Submitted filename: Response to Reviewers.docx [file pone.0338383.s001.docx]

**Dear Editors of PLOS ONE,**

We would like to thank the previous reviewers and the editor for their insightful comments and valuable suggestions, which have helped us to improve the quality of our manuscript significantly. The current manuscript PONE-D-25-41210 is a resubmission of the previous manuscript PONE-D-25-06205 that we submitted to "Plos One" before. We carefully considered the provided feedback and made significant revisions to the manuscript PONE-D-25-06205 based on the feedback.

The reviewer #1's comments were positive, but she seemed to be dissatisfied with the writing structure, language expression and excessive word count of the manuscript. Therefore, we carried out extensive structural optimization and language refinement on the previous manuscript PONE-D-25-06205, which significantly enhanced the readability of the manuscript.

Reviewer #2 rejected the publication of our manuscript. We carefully read the comments from Reviewer #2 and found that his main concerns were the lack of justification for sample size, the insufficient familiarization period for performance tests, and the incomplete description of the training program. In response to this, our team re-evaluated the previous manuscript PONE-D-25-06205, the research protocol, ethical review, and clinical trial registration, among other relevant documents. We discovered that during the writing of the manuscript, due to the length, many details were not described in detail. For example: the description of sample size estimation, detailed conceptual descriptions, and detailed descriptions of experimental blinding, etc. Additionally, we believe that our research is beneficial for improving the athletic performance of young athletes, which is meaningful for competitive sports. Surprisingly, Reviewer #2 also expressed the same view as us in his comments and revision suggestions. After carefully considering Reviewer #2's suggestions regarding our manuscript, we believe that the manuscript can be revised. Therefore, we are very grateful to Reviewer #2 for providing us with very detailed and valuable suggestions. Based on this, our team made significant revisions to the manuscript PONE-D-25-06205.

**Below is a detailed point-by-point response.**

**Reviewer #1:**

**Comment 1:** Article was good but not as such congerence or relationship between sentence some type of vague but it's okh. And article need the improvement in almost all parts of article including abstract introduction discussion conclusion rationale and concentrate on the tables if not necessary than remove and word count is tooo much excess and focused on reference the same pattern is not followed in all parts of articles.

**Response:** Firstly, we carried out extensive structural optimization and language refinement on the manuscript PONE-D-25-06205, significantly enhancing its readability. Secondly, the images 1-5 have been beautified. The tables 3-6 have removed overlapping indicators and unnecessary data, enhancing the overall appearance and simplicity.

**Reviewer #2:**

**Comment 1:**

Abstract

(1) Background for study is missing in abstract.
(2) The abstract lists too many test variables which burdens readability. Consider consolidating under broader categories
(3) Key outcome statements (e.g., “significant improvements”) are not backed by quantitative data or p-values.
(4) Pte-stretch augmentation percentage seems to be a error.
(5) The statement on perceived fatigue lacks clarity.
(6) Use only standard or technical Abbreviations

**Response:**

(1) As suggested, we have supplemented the background section of the abstract (Lines 12-15)
(2) We summarized the variables of the test indicators and classified them as significant or insignificant, which made the presentation of the results more clear. (Lines 18-20)
(3) We classified the significance of the test indicators and supplemented with quantitative data or p-values.
(4) We have removed "Pte-stretch augmentation percentage"
(5) In the abstract, we indicated the use of the perceived exertion scale rating for fatigue monitoring. However, considering the brevity of the abstract, it is not clear whether a more detailed explanation is necessary. Therefore, we have chosen to retain the original expression. (Lines 20-21)
(6) We have removed all non-standardized abbreviations from the abstract section, but have retained the descriptions of the experimental group and the control group (FCMT group and CT group).

**Comment 2:**

**Introduction**

(1) The introduction is Overextended Background on PAP and Su detracts from the central focus on French Contrast Method Training (FCMT).
(2) There is a major issue in the Conceptual Clarity Between CT and FCMT; since the study is a direct comparison of both, clearly define their theoretical and practical differences early on with sharper contrast—particularly in structure, load intensity, and progression logic.
(3) You have not provided the rationale for including the adolescent badminton players
(4) While rule changes and match structure are mentioned (e.g., 1:2 play/rest ratio), there is no clear connection to why lower limb explosive endurance is crucial for these players.
(5) The statement that the adaptation timelines of CT and FCMT are “yet to be defined” lacks references or theoretical support. Cite relevant physiological or training adaptation literature to anchor this claim.
(6) Rewrite the research questions into objectives
(7) A lot many statements are written in generalized form and lacks referencing.
(8) The contrast training definition and its impact is not well defined in introduction.

**Response:**

(1) According to the suggestions, we removed Su's examples and shortened the excessive description of PAP, and provided a more detailed description of the conceptual and essential differences between the French Contrast Method Training and the Complex Training. (Lines 37-58)
(2) Same as above.
(3) The rationale for choosing youth badminton is described in the "Participants" section. Firstly, in the introduction, we have already mentioned that explosive power and its endurance are the key factors for winning in badminton, which is why we chose this sport. Secondly, previous similar studies have found that French Contrast Method Training is beneficial for improving the explosive power of adult athletes. However, the current research field of French Contrast Method Training lacks exploration of the adolescent group. Moreover, previous studies have shown that physiological maturity can affect the training responses of the two types of training (French Contrast Method Training and Complex Training), which may cause different training responses in adolescents during their growth and development period. Therefore, we chose young badminton players as the subjects. (Lines 60-72) (Lines 114-116)
(4) We have retained the descriptions of rule changes and the competition structure, and have added the importance of lower body explosive power and its endurance in badminton. (Lines 60-70)
(5) This part was described in the "Monitoring Indicators & Processes" section. We chose the RPE scale for fatigue monitoring, with the main purpose of reflecting the adaptation of the two groups of subjects to the corresponding training methods throughout the intervention period. We have also previously cited relevant physiological or training adaptation literature to support the rationality of the methodology. (Lines 207-211)
(6) According to the suggestion, the research question has been changed to objectives. (Lines 77-80)
(7) We carefully examined the sentences and citations in the introduction section. We did find cases where the language was overly general and the citations were insufficient. To address this, we refined the introduction section and added new citations of 13 additional sources, thereby enhancing the rationality and scientificity of the introduction.
(8) We are not sure whether "contrast training" in the comments refers to the French contrast group training or something else. Therefore, we did not make any modifications based on this comment. If it refers to the French contrast group training, we have provided a detailed description of its definition and impact in Lines 37-45.

**Comment 3:**

Methods

(1) No sample size calculation or power analysis is reported. For a randomized controlled trial (RCT), a priori determination of sample size is critical to ensure sufficient power to detect meaningful effects. The authors should specify the expected effect size, alpha level, and power used for estimation, or at least cite similar studies to justify the chosen sample size (n=10 per group).
(2) While exercises and loads are mentioned, training progression, periodization, and intensity control across the 8 weeks are not clearly defined. Were any adjustments made based on participant progress or fatigue? Was load reassessed midway?
(3) Also, volume equality between FCMT and CT groups is mentioned, but not shown quantitatively. A tabular summary of weekly sets × reps × load would enhance clarity and reproducibility.
(4) There is no mention of a familiarization period for the performance tests (e.g., 5-0-5, squat jumps, CMJ, LEEE).
(5) Although described as "single-blind," the manuscript does not specify who was blinded—participants, assessors, or trainers? Clarify who remained blinded and how bias was minimized during assessments.
(6) The manuscript briefly mentions the “random number table method,” but does not describe how allocation concealment was ensured. Was randomization conducted by an independent party? Was allocation concealed until assignment?
(7) The authors should explain how external factors were standardized or monitored.
(8) While ethics approval and parental consent are mentioned, no mention is made of adverse event monitoring or safety assessments for adolescent participants.
(9) The manuscript does not identify who designed or validated the training protocols for FCMT and CT. Was it developed by certified strength and conditioning professionals, sports scientists, or adapted from previous peer-reviewed protocols? For transparency and scientific rigor, the authors should specify whether the protocol was adapted from established literature (e.g., Dietz’s triphasic training model) and whether any expert supervision or external validation was involved in planning or overseeing the intervention.
(10) Additionally, it is unclear who delivered the training (e.g., certified trainers, researchers) and what steps were taken to ensure fidelity to the protocol across all sessions.
(11) Figure 1 does not follow CONSORT flow diagram standards for randomized trials.
(12) The terms “control group” and “experimental group” are used, but it's better to use more descriptive labels (e.g., “CT Group (n=10)” and “FCMT Group (n=10)”) directly in the figure to enhance reader clarity.
(13) The manuscript mentions that participants were included if they had an FMS score above zero and an over-the-shoulder squat score of at least 2. However, this inclusion threshold requires clarification: (1) Since the total FMS score ranges from 0 to 21, a score “above zero” is an extremely lenient criterion and does not reflect functional competency. Please clarify whether there was a minimum total FMS cutoff which is more commonly used to indicate acceptable movement quality and reduced injury risk. (2) This seems to be a subcomponent of the FMS (likely the deep squat test), but the rationale for choosing this specific movement and cutoff is not provided. Please explain why this test was prioritized and whether it was used to ensure movement safety or screen for asymmetries prior to training. (3) The manuscript does not report the mean or range of FMS scores for the included participants for both the groups; and not justified the homogeneity of the sample.
(14) The manuscript occasionally uses the term “compound training”, which appears to be a misnomer or an unintentional substitution for “complex training”
(15) The study includes a large number of outcome variables: CMJ, SJ, DJ, DJ-RSI, EUR, PSAP, SLJ, 10m sprint, 5-0-5 test, LEEE, and 1RM strength. While comprehensive, the rationale for selecting each test is not adequately provided, especially when some variables (e.g., CMJ vs. DJ, or EUR vs. PSAP) are closely related or overlapping in measuring SSC performance.
(16) The use of “velocity dispersion during 15 reps of half squats with 20kg” as a marker for explosive endurance (LEEE) is innovative but not commonly used or validated in published literature. Justify the 20 kg load and number of repetitions chosen
(17) The test schedule in Figure 2 lacks specificity. It should indicate Which tests were conducted on which day, recovery periods between tests, whether testers were blinded
(18) The rationale for the order of test administration is not described. Test order can influence outcomes (e.g., fatigue from earlier tests affecting later ones). Clarifying whether the sequence was randomized or fixed would improve the figure’s utility.
(19) Neither Figure 1 nor Figure 2 is sufficiently cross-referenced in the main text. The Methods section should include to guide the reader.

**Response:**

(1) Before the research was conducted, we had estimated the sample size required for the experiment. However, considering the excessive length of the manuscript, we decided to omit this part. But we realized that this was not advisable. In the new version of the manuscript, we re-added this part (Lines 105-109). The sample size required after pre-estimation was 24, but the final sample size we included was 20, thus there was a shortage of sample size. For this inevitable situation, we used Hedges' g value as the effect size to compensate for the statistical power (Lines 224-225). Additionally, the experimental subjects we chose were scarce, so we adopted the reviewer's suggestion and added references to similar studies to prove that the selected sample size (n=10) was feasible.
(2) In the Study Design section, we have already mentioned that during the 8-week intervention period, both groups underwent training with a fixed load (the same load weight, repetition count, and number of sets) (Lines 98-100). There was no adjustment of the load based on the participants' progress or fatigue level during the process. The aim was to observe the adaptation trend of the participants to the intervention program throughout the entire experimental period. This was also mentioned in the previous version.
(3) In Table 1, we have already listed the load weight, repetition count and number of sets for each group, which helps to enhance the clarity and reproducibility for the readers.
(4) In the "Study Design" section, we have already mentioned that "All subjects were accustomed to both interventions and testing procedures prior to the intervention period." (Lines 88-89). The testing procedures here include 5-0-5, squat jumps, CMJ, LEEE, etc. This content was also mentioned in the previous version of the manuscript.
(5) In the "Study Design" section, we stated that this was a single-blind study and described that "the participants were unaware of the intervention purpose and group assignment" (Lines 83-85). Considering that professional fitness coaches need to be very familiar with the intervention content and guide the participants, we did not impose blinding on the professional fitness coaches. Additionally, to minimize the bias in the assessment process, we set up two test days (T1 and T2), and the test sequence, participant and test personnel organization remained consistent throughout. For a detailed description, please refer to Lines 141-148 of the manuscript.
(6) We used the envelope method for group concealment, but it was not described before. We added the details in Lines 90-92.
(7) In Lines 123-126, we described that a standardized 20-minute warm-up consists of dynamic stretching and muscle activation, while a standardized 10-minute relaxation involves static stretching.
(8) During the training process, each group had two professional fitness coaches stationed on either side of the barbell to provide protection and prevent any safety incidents. Furthermore, during the selection of the subjects, we conducted a screening of their medical history and evaluated the safety of the squatting movement pattern to prevent any injuries.
(9) The training plans adopted in these two experiments were jointly designed by the researchers of the experiment and two professional and certified fitness coaches. The training plans were adapted from the study by Hernandez et al. We made some additions to the manuscript (Lines 126-128).
(10) During the training session, 3 researchers were present to supervise the progress of the training plan. Each group was equipped with 2 professionally certified fitness coaches to ensure the quality of the movements and provide emergency protection.
(11) We have optimized and modified Figure 1.
(12) According to the suggestion, we will replace "control group" in the chart with "CT Group" and "experimental group" with "FCMT Group"
(13) During the screening process of the subjects, we only used one sub-component of FMS, namely the over-the-head squat, for the screening purpose, in order to check whether the subjects had any obstacles in their squatting movement patterns. We believe this is necessary because it is crucial for the quality of the implementation of our intervention plan. This approach was also adopted in our previous study (Reference: Huang R, Zhang M, Huang L, Chen Z, Mo Y and Gao Y (2023) Effects of lower-extremity explosive strength on youth judo athletes adopting different types of power-based resistance training. Front. Physiol. 14:1065036. doi: 10.3389/fphys.2023.1065036). Furthermore, the overhead squat test is only used for the screening of the participants. The full score for this project is 3 points, but our selection criteria are at least 2 points. However, after our observation, all the test subjects' scores were 3 points. This might be attributed to the agility of badminton players. Therefore, we did not choose to include the average score of the two groups in the basic characteristics of the test subjects.
(14) After our investigation, all the incorrect spellings of "compound training" have been corrected to "Complex Training".
(15) Although our study covered a wide range of evaluation indicators, for each indicator, we have explained the reasons for the choice or provided relevant references (Lines 153-156), (Lines 165), (Lines 168), (Lines 171), (Lines 175), (Lines 178), (Lines 183), (Lines 201), (Lines 171), and (Lines 209-211). Since we took into account that the influencing factors of athletes' explosive power are multi-dimensional (possibly the increase in maximum strength, possibly the improvement in eccentric utilization rate, or the enhancement of power, etc.), and both training methods can effectively develop the power ability of athletes. If only one type of explosive force assessment method is used, it may not be possible to accurately observe the reasons for the difference in performance between the two training methods. Just as our research results show, the French Contrast Method Training has advantages in aspects such as response-power, agility, sprint speed efficiency, and explosive endurance. Without adopting this method, we would not be able to observe these characteristics. Ultimately, after our discussion, we adopted some of the suggestions mentioned in the comments. Considering that there is unnecessary overlap between EUR and PSAP, as well as between SLD and the jump test, we have decided to remove PSAP and SLD.
(16) This might be a description error (describing the average value as weight), as we used 15 repetitions of 30% 1RM weight for squatting to assess the explosive endurance of the subjects (Lines 193-199). The choice of 30% 1RM and 15 repetitions mainly refers to the load corresponding to the development of speed-intensity in the force-velocity curve. Considering that the squatting and training content have the same movement pattern, we chose this assessment method to avoid deviations caused by different movement patterns. Although this method is rarely found in published literature, we have provided similar references to support it. Therefore, we believe that this method is theoretically feasible. The reasons are as follows: Firstly, similar action patterns reduce the deviation of results. Secondly, we adopted linear sensors for speed monitoring, and the principle is similar to that of resistance training based on "velocity loss". Furthermore, statistically, the standard deviation of a set of data represents the degree of dispersion of that data. By observing the standard deviation of the speed values of 15 squats, we can determine whether the subjects can maintain a high level of explosive power. However, this is only possible if each squat is performed with maximum effort. Therefore, we organized professional fitness coaches to provide unexpected protection while encouraging the subjects verbally.
(17) We have beautified and modified Figure 2 to make it more intuitive. The grouping of the testers is concealed, so they are never aware of the grouping situation.
(18) We mentioned the basis for our test scheduling order in Lines 144-145. In Lines, it was also mentioned that the test sequence and personnel arrangement remained consistent throughout (Lines 148).
(19) According to the suggestion, we have added the references to Figure 1 and Figure 2 in the methods section (Lines 110) (Lines 142).

**Comment 4:**

Results

(1) The Results section is overloaded with variables
(2) Figure 4 and Figure 5 are redundant consider merging or removing one for conciseness.
(3) Effect sizes are reported but not interpreted
(4) Figure 3 lacks error bars and clear session labelling revise to improve visual clarity of fatigue trends.
(5) Between-group change comparisons are not summarized in a single table add one for quick interpretation.
(6) Inconsistent use of statistical terms (p-values, MBI, CI)—standardize and explain thresholds.

**Response:**

(1) According to the suggestions, we have optimized the content and structure of the results section, eliminating excessive variables.
(2) We removed the original Figure 4 as per the suggestion, because we considered that our main objective was to compare the advantages and disadvantages of the two training methods. Therefore, we retained Figure 5.
(3) Based on the suggestions, we have added the necessary explanations of effect sizes (Lines 263-266).
(4) We attempted to add error bars to Figure 3, but the result was not satisfactory. Especially in Training Sessions 1, 2, 3, 8, and 9, it became even more blurry. However, to make it more intuitive and clear, we added the average RPE score of each session for each group at the bottom of the curve.
(5) Figure 6 illustrates the differences between the groups.
(6) In the new version of the manuscript, we removed MBI because we believed that the controversy surrounding this statistical method was too significant. MBI is not necessary, and the remaining statistical methods can also make the data more intuitive. Additionally, according to the suggestions, we standardized the expressions of statistical terms such as p-values and confidence intervals.

**Comment 5:**

Discussion

(1) The discussion reiterates results without deep mechanistic insight add physiological interpretation.
(2) Claims about neuromuscular excitation are speculative support with literature or qualify accordingly.
(3) Prior studies are cited selectively include contrasting evidence for balanced discussion.
(4) The Su Bingtian example fits better in the introduction remove or move to improve focus.
(5) No limitations of the outcome measures themselves are acknowledged address their sensitivity or validity.
(6) Training progression and long-term application are not discussed consider implications for periodization.
(7) SSC-related jargon may confuse non-specialists simplify or define key terms.
(8) The discussion lacks athlete-centered practical recommendations add actionable takeaways for coaches.

**Response:**

(1) We reemphasized the purpose of presenting the results, which was to highlight the specific differences between the two training methods and to enable readers to better understand the subsequent discussion section. However, we also realized that the old manuscript had overly detailed descriptions of the results. Therefore, in the new manuscript, we have streamlined the presentation of the results (Lines 276-278) and deepened the physiological interpretation.
(2) The claims about neuromuscular excitation are inferred by combining experimental results, literature, and related theories. Firstly, in terms of literature, Cal Dietz et al. (Lines 331-334) conducted research and proposed that the French Contrast Method Training has advantages in terms of neural-muscular efficiency and coordination. Secondly, the experimental results reflect the advantages of the French Contrast Method Training in enhancing reactive power, agility ability, and SSC utilization ability, which are all highly related to the neural-muscular system, but this is an indirect reflection. Moreover, the training structure of the French Contrast Method Training is conducive to maximizing the post-activation potentiation effect, thereby reducing the sense of autonomic fatigue and optimizing the power output of each group of French contrast group training (Lines 283-307). The results of fatigue monitoring also support this inference. Additionally, we have cited relevant theories about the physiology and metabolism of the neural-muscular system for verification. The specific content can be read in Lines 308-326.
(3) We understand this confusion, but we did not selectively cite previous studies. This is because there is a lack of research on French Contrast Method Training, and the results are consistently favorable. From our observation, there are almost no comparative studies between French Contrast Method Training and Complex Training. Although complex training is effective and widespread in developing athletes' explosive power. We can only use literature on intermittent training with similar training structures to support our discussion on the gain in explosive endurance.
(4) According to the suggestion, we removed Su's example because it was intended to highlight the importance of PAP, but it seemed to be of no help in focusing our research.
(5) In the discussion section (Lines 355-363), we elaborated on the limitations of our research.
(6) In the "Discussion" section, from Lines 284 to 305 and Lines 314 to 317, we discuss "Training progression" by analyzing the training structure. In Lines 345 to 348, we discuss (Lines 365 to 375) the long-term application of periodization.
(7) When SSC first appeared in the main text (lines 175-176), a brief explanation was given for it.
(8) As the comments indicate, in the previous version of the manuscript, there were too few practical suggestions centered on the athletes and too few operational suggestions from the coaches. In the new version of the manuscript, we have placed greater emphasis on these two aspects (Lines 366-376).

**We sincerely appreciate the dedicated work of the editors and reviewers, who provided us with a great many valuable suggestions. We hope the revised manuscript meets your expectations and look forward to your positive consideration. If you have any questions, please feel free to contact me at any time.**

**Sincerely,**

Yuhua Gao, PhD

Guangzhou sport University, China

Email: hry13066290079@gmail.com
