# Supplementary material for: Comparative effects of French Contrast Method vs. Complex Training on explosive power and its endurance in youth badminton athletes
Source: PLoS One. 2025 Dec 5;20(12):e0338383. doi: 10.1371/journal.pone.0338383 (PMC12680223; doi:10.1371/journal.pone.0338383)
Supplement: Attachment — Submitted filename: Response_to_Reviewers_auresp_1.docx [file pone.0338383.s002.docx]

#### Response to Reviewers

**Manuscript ID: PONE-D-25-41210**

**Journal: PLOS ONE**

**Title: Comparative Effects of French Contrast Method vs. Complex Training on Explosive Power and its Endurance in Youth Badminton Athletes**

**Data DOI: 10.6084/m9.figshare.30349558**

**Dear Editors of PLOS ONE,**

We sincerely thank the Academic Editor and both reviewers for their thoughtful critiques and the opportunity to submit a revised manuscript. In response,we implemented revisions to improve theoretical framing, methodological transparency, and interpretive balance. Key changes include: (1) strengthening the theoretical framework in the Introduction and Discussion; (2) explicitly justifying methodological choices (e.g., fixed loads; differing exercises) and acknowledging their implications; (3) integrating all suggested citations; (4) tempering claims to align strictly with the data and study limitations; and (5) updating data and figure compliance. The dataset has been re-uploaded to Figshare (DOI: 10.6084/m9.figshare.30349558), and figures were checked with PACE.

**Below is a detailed point-by-point response.**

**Response to Reviewer #1:**

We sincerely thank Reviewer #1 for their insightful and constructive comments, which have been instrumental in strengthening our manuscript. First, concerning the use of different exercises between the FCMT and CT groups, we acknowledge this as an inherent aspect of comparing these distinct training methodologies. The French Contrast Method Training is structured around a specific four-exercise sequence designed to systematically exploit post-activation performance enhancement (PAPE), which necessarily involves different exercises and loading patterns than the paired-exercise structure of traditional Complex Training. We recognize that this difference introduces a potential confounding variable. Accordingly, we have clarified the rationale for our exercise selection in the Methods and explicitly acknowledged this limitation in the Discussion, recommending that future studies match exercises to isolate the effect of training structure. Second, we agree that the tone in the original Introduction and Discussion could be perceived as overly promotional. We have thoroughly revised these sections to adopt a more balanced and scientific perspective. This includes tempering claims about FCMT, citing relevant literature such as the review by Cormier et al. to provide context, and using more cautious language (e.g., "may," "suggest") when interpreting results. We have also emphasized outcomes where both training methods were equally effective to ensure a balanced presentation. Our point-by-point responses below detail all specific changes made in the manuscript.

**Comment 1:**

Introduction:

(1) Line 35 – this first sentence also needs some commentary around the importance of power. It is really the need to improve both simultaneously that has created the interest in contrast training methods.
(2) Line 37 – building on the prior point, it would be good to expand on this. The reasons it is becoming popular is not because it is the best way t improve strength. It is because it is suggested to offer an effective way to improve strength and power simultaneously.
(3) Line 37 – it would also be good to introduce complex training more broadly before describing French contrast and complex contrast (which is what you are examining). The review paper by Comier is a great place to start (DOI: 10.1007/s40279-022-01715-x).
(4) Line 38 – you need to explain what PAP is. It would also be more appropriate to describe it as post-activation performance enhancement (PAPE) as that is what you are talking about.
(5) Line 41 – need to temper your language here. While there is evidence to suggest that the PAPE response does improve performance in the short term, there is limited to suggest that these modes of training offer any additional advantages in the long term. It is “theorised” to enhance performance more than traditional training methods.
(6) Line 51 to 56 – this reads like an advertisement. All of this is theoretical. Suggest removing and/or tempering your language and accurately representing the research on FCT, and removing buzzwords.
(7) Line 68 – remove “centripetal and centrifugal”
(8) Line 73 – remove “(i.e., complex training)” as neither of these studies looked at complex training.
(9) Line 74 to 75 – this citation is not appropriate for this statement, and it is only partially accurate. A Delphi study of elite strength and condition coaches by Luders et al., 2024 (https://doi.org/10.1177/17479541241272256) suggested that it is used because it is time efficient. A follow up intervention found complex training to be equally as effective at improving athletic performance as normal strength and power training, albeit took less time to complete (https://doi.org/10.1519/JSC.0000000000004888). A more balanced discussion of the potential benefits of these types of training methods supported by the above studies would be better here.
(10) Line 78 – This aim/objective is different to your trial registration, where you explicitly state that you expect FCMT to be better. Please revise in line with the registration.

**Response:**

(1) We have restructured the opening section of the introduction based on the reviewers' suggestions. The revised content first explicitly defines badminton as a multidirectional explosive sport, noting that outstanding performance depends not only on strength and power but also on highly efficient neuromuscular coordination. Building on this foundation, we further emphasize that the distinct physiological demands of strength and power during training adaptation make their simultaneous development challenging. This naturally leads to the introduction of the Post-Activated Performance Enhancement (PAPE) mechanism and its derivative methods, such as Complex Training (CT) and the French Contrast Method of Training (FCMT). (Lines 37-53)
(2) Synchronized with the modifications to (1).
(3) We have incorporated the reviewers' suggestions by providing a broader introduction to complex training before describing the two training methods. After carefully reviewing the study by Comier et al., we found it effectively explains the concept and principles of wind complex training, and have therefore cited it. (Lines 45-47)
(4) Indeed, describing it as PAPE would be more appropriate, as most of our test metrics are used for performance evaluation. We have explained PAPE in the text. (Lines 43-45)
(5) Indeed, in our original manuscript, we overemphasized the description of FCMT and became overly “theoretical” when discussing long-term effects, lacking sufficient evidence. Therefore, we have revised our wording. (Lines 50-52)
(6) We have removed the previous version and made adjustments to the description of FCMT. (Lines 50-52)
(7) We have removed it.
(8) We have removed it.
(9) We sincerely thank the reviewers for providing us with two constructive references. We have reviewed our previous citations and found that we did not sufficiently substantiate the claim that “its effectiveness has been widely recognized by coaches.” Therefore, we have incorporated the two references suggested by the reviewers, which provide robust evidence for the time efficiency and performance benefits of CT from both the coaches' and athletes' perspectives. (Lines 64-66)
(10) We have adjusted the description of the objectives based on the trial registration. (Lines 69-71)

**Comment 2:**

Methods:

(1) Line 88 – can you please detail how they were “adapted” to both interventions?
(2) Line 89 – this method of randomisation is different to that described in the pre-registration. Please revise or add a section title “deviations from pre-planned protocol” to highlight these deviations.
(3) Ine 135 – you need to justify your exercise selection here. Why were they different? Also need to provide some explanation on how they were performed (tempo etc) so that any differences are really clear.
(4) General comment – it also looks like a few tests you looked at were not included in your trial registration. These should also be notes when describing other deviations.
(5) General comment – in your registration you state the data will be made publicly available. It should be noted here with the link to the repository provided.
(6) General comment – statistical analysis. There is a huge number of analyses conducted here. You should do a post-hoc correction for multiple comparisons, as some of the between group differences may just be through chance due to your small sample and large number of comparisons.

**Response:**

(1) We acknowledge that the term “adapted” was used incorrectly; our intent was to convey ‘familiar’ (lines 79-81). The wording in this section has been revised, and the training details are described in the “Training program” section at lines 143-144.
(2) We have modified the pre-registered randomization method to ensure consistency. (Lines 82-88)
(3) We restructured the description of the “Training program” section to clarify the rationale behind exercise selection. We explained why different exercises were chosen by discussing the fundamental differences between the two training approaches (organizational structure and maximizing PAPE) (lines 123-132). Additionally, we supplemented details on exercise tempo and set execution (lines 139-144).
(4) We have removed two metrics from the previously submitted manuscript: the standing long jump and the Pre-stretch augmentation percentage (PSAP). This addresses the previous reviewer's suggestion that our test metrics overlapped. Between the standing long jump and the vertical jump, we selected the vertical jump due to its superior ability to reflect lower-limb dynamics. Additionally, between the Eccentric Utilization Ratio (EUR) and PSAP, we selected EUR because it is more oriented toward assessment, whereas PSAP is more suited for diagnosing personalized training protocols.
(5) Our data is provided in the “Date Availability Statements” section. “The data that support the findings of this study are available in Figshare at <http://doi.org/10.6084/m9.figshare.30349558.”>
(6) Given the small sample size, we adopted the reviewers' suggestion to perform post-hoc corrections for multiple comparisons. Tukey's method was used for post-hoc tests when data followed a normal distribution, while the Dwass-Steel-Critchlow-Fligner method was applied when data did not follow a normal distribution. The corrected results did not negate the original conclusions. (lines 237-240)

**Comment 3:**

Results:

(1) General comment – would be good to report mean and SD for session adherence here somewhere as well.
(2) Line 239 – remove the word autonomic from this section. You are simply assessing perceived effort. Moreover, none of this was compared statistically, so this cannot really be stated. The figure looks as if the changes/differences are small, and unlikely meaningful. Suggest removing commentary around the differences.?

**Response:**

(1) Regarding session adherence, all participants in the trial completed all training and testing sessions without any absences or dropouts. This point is also mentioned in lines 247-249. (No subjects reported adverse effects due to injury or early withdrawal from the study over the course of the 8-week study, resulting in a total of 20 subjects (10 in the FCMT group and 10 in the CT group) included in the metrics data analyzed statistically.)
(2) We have removed the term “autonomic.” However, we believe analysis of fatigue monitoring is necessary. Although we did not perform statistical comparisons here, the plotted “average change curve of RPE scale indices after 16 training sessions for both groups” (Figure 3) clearly shows that the difference in perceived exertion post-training between the two groups increased after the 9th session. We have also re-added error bars to Figure 3, which allows for more intuitive observation of this point. (lines 254-261)

**Comment 4:**

Discussion:

(1) General comment – the discussion focuses on FCMTs “superiority” by a bunch of unsubstantiated mechanisms (for example, “neuromechanical advantages across the force–velocity spectrum,”) but no direct neural or mechanical data was collected. This is just speculation. As such, the language throughout the discussion needs to reflect that these are potential explanations rather than conclusions.
(2) General comment – the outcomes whereby there were no between-group differences is downplayed. Both methods seem equally effective for maximal strength and concentric, which should be mentioned in more detail.
(3) Line 286 – not sure if “plate” is the correct word here. Maybe stage or section?
(4) Line 287 to 291 – so much of this is non-scientific and just buzzwords. I would suggest highlighting some of the differences between the interventions as a way to discuss some potential difference sin outcomes, rather than just making statements about the hypothetical benefits of FCT.
(5) Line 357 – another limitation that need to be added: A) The exercises within the interventions were different. As a result, it is unclear whether the differences observed are due to the different training structures, or simply the different exercises.

**Response:**

(1) We agree with the reviewer's perspective. For a series of unproven mechanisms, we have adjusted the wording of our discussion by using terms such as “potentially” and “seem to” to reflect that these represent potential explanations rather than definitive conclusions.
(2) We greatly appreciate the reviewer's insightful suggestion. This was indeed an oversight on our part. We have added a paragraph describing this in lines 387–398.
(3) Our original intent was to include a section. However, considering that this part of the description was overly subjective and lacked supporting evidence, we deleted it and rewrote the part. (lines 298-324)
(4) As stated in (3), we rewrote this section because we considered it overly subjective and lacking direct evidence. We first explicitly clarified that the efficacy difference between FCMT and CT stems from their distinct training structures and activation methods, establishing a comparative foundation by citing methodological details. Subsequently, we conducted a multidimensional mechanism analysis: FCMT's load progression sequence provides more comprehensive stimulation of the force-velocity curve (referencing Dietz C et al.'s “Triphasic training: A systematic approach to elite speed and explosive strength performance”), aligning with modern joint coordination training principles (incorporating Reviewer 2's suggestions) to enhance kinetic chain efficiency and movement economy; simultaneously, the external load reduction strategy delays neuromuscular fatigue, supported by subjective fatigue data. Subsequently, by linking the training method to outcome measures such as reaction force and SSC efficiency through the relationship between the isometric contraction characteristics of static squats and the enhancement of rate of force development (RFD), and by citing literature to establish the theoretical connection between RFD and lower-body explosive qualities. Finally, multiple athlete studies are referenced to substantiate the argument, completing the closed-loop reasoning from methodological differences → physiological mechanisms → empirical results → theoretical elevation. (lines 298-324)
(5) This is necessary, so we have added “D)” to the restrictions section. (lines 380-381)

**Comment 5:**

Conclusion:

(1) Line 385 – suggest replacing “can” with “may” as you don’t know the reason.
(2) Would also be good to add something like “Future studies should match exercises and volume between conditions to isolate the effects of training structure.”
(3) Table 1 – the exercises in this table are different to that in the methods. Please revise for consistency and clarify.
(4) Figure 3 – add standard deviations to each time point.

**Response:**

(1) Based on the feedback, we have made adjustments to the wording.
(2) We have added this suggestion in lines 397-399.
(3) We reviewed the statements in Table 1 and the Methods section and made revisions to the wording in the Methods section. (lines 133-138)
(4) We have added error bars to Figure 3.

**Response to Reviewer #2:**

We sincerely thank Reviewer #2 for their thorough and insightful evaluation and for acknowledging the improvements made to the manuscript. Their comments on methodological weaknesses were particularly valuable in enhancing the scientific rigor and clarity of our work. Regarding the Fixed Training Load Protocol, we acknowledge the valid concern that using a fixed load over 8 weeks deviates from the principle of progressive loading. We clarify that this was a deliberate choice to standardize the intervention and observe adaptation trends to the training structures themselves, without the confound of load progression. We agree this limits external validity, and we have therefore explicitly acknowledged it as a primary limitation in the Discussion. Our conclusions have been tempered to state that the findings are specific to the fixed-load context, and we recommend that future studies adopt phased loading protocols to further explore optimal training ranges. Regarding the Validity of the LEEE Test, we agree that this novel test requires robust validation. In response, we have strengthened the methodological rationale in the Methods by citing relevant literature on explosive endurance and clarifying the test's design logic, including movement selection, load, and repetition adjustments tailored to badminton biomechanics. Regarding the Minor Weaknesses, all have been addressed: the Methods now provide a clearer participant screening description; standard error bars have been added to Figure 3; and the small sample size limitation is acknowledged, with additional statistical safeguards (e.g., post-hoc corrections) applied where appropriate. Our point-by-point responses below detail all specific changes made in the manuscript

**Comment 1:**

Abstract:

(1) Page 8, Lines 124-126: The statement "...FCMT showed a delayed but more substantial performance enhancement after 4 weeks, with lower cumulative RPE scores" could be misinterpreted. The RPE scores for FCMT were consistently lower throughout, not just cumulatively . The key finding is the differing adaptation timelines, which should be stated more clearly.

**Response:**

(1) As suggested, we made the changes on lines 24-26.

**Comment 2:**

Introduction:

(1) Page 11, Lines 185-186: The introduction could be strengthened by providing a more detailed biomechanical rationale for why explosive power is critical in badminton. Citing recent work on muscle synergy and neuromuscular coordination during key movements like the overhead smash would provide a stronger foundation for investigating advanced training methods that target these qualities. It is recommended to include the following reference:[Tajik R, Dhahbi W, Fadaei H, Mimar R: Muscle Synergy Analysis During Badminton Forehand Overhead Smash: Integrating Electromyography and Musculoskeletal Modeling. Frontiers in Sports and Active Living 2025,

**Response:**

(1) This is an excellent idea. We have incorporated relevant content into the introduction and cited the recommended literature. This source provides detailed biomechanical foundations for specialized badminton techniques. This better positions us to introduce subsequent complex training and French contrast method training. (Lines 39-40)

**Comment 3:**

Methods:

(1) Page 14, Lines 258-259: As stated in the major weaknesses, the sentence "Both groups were trained with a fixed load... during the 8-week intervention period" details a significant methodological flaw . The rationale to "observe the trend of subjects' adaptation" does not justify violating the fundamental training principle of progressive overload.
(2) Page 20, Lines 456-458: In the Maximum Strength Test subsection, the direct measurement protocol for 1RM is described . To bolster the methodological rigor, it is recommended to cite recent literature that validates or discusses protocols for direct 1RM testing in athletic populations. The following reference would be appropriate. [Dhahbi W, Padulo J, Russo L, Racil G, Ltifi M-A, Picerno P, Iuliano E, Migliaccio GM: 4-6 Repetition Maximum (RM) and 1-RM Prediction in Free-Weight Bench Press and Smith Machine Squat Based on Body Mass in Male Athletes. Journal of strength and conditioning research 2024.]
(3) Page 22, Lines 502-503: The explicit statement that the Maximum Strength and LEEE tests were not included in reliability assessment is a notable weakness for the LEEE test . While understandable for a 1RM test, the lack of reliability data for a novel primary outcome measure is problematic.

**Response:**

(1) We fully understand the reviewers' legitimate concerns regarding the fixed training load, as it deviates from the principle of progressive overload. We clarify that this was a deliberate choice made to standardize the intervention and observe subjects' adaptation trends to the training regimen, thereby avoiding interference from load progression. Furthermore, the timing of subjects' adaptation to the training regimen was an outcome derived from the study; we did not know the answer prior to conducting the research. Our concern is that altering loads without a precise adaptation timeline could also introduce some bias into the study results. However, we fully acknowledge that this limits the study's generalizability and have explicitly listed it as a major limitation in the Discussion section. We have carefully adjusted the conclusion statement to explicitly indicate that the findings apply only to fixed-load scenarios. We recommend that future studies employ phased loading protocols or adjust loads based on the adaptation timepoints identified in this study to further explore optimal training intervals.
(2) This is an excellent suggestion. We have reviewed the previous references and added this recent literature to further strengthen the rigor of the methodology. (Lines 212-213)
(3) We fully understand the reviewers' concerns. The lack of reliability analysis for innovative test metrics is indeed a significant shortcoming. Our concern is that the continuous power output test caused a substantial decline in participants' physical condition, which greatly impacted the accuracy of the second test. We retrained participants on the testing procedures before the post-experiment assessment, during which the LEEE test was administered twice. We observed substantial differences between the second and first results. However, we did not include the training session data in the dataset, as it did not constitute a formal testing procedure. Consequently, the LEEE test was not administered a second time during the formal post-test phase, and thus no reliability analysis was performed. This decision is explained in the “Statistical Analyses” section. (Lines 233-234)

**Comment 4:**

Discussion:

(1) Page 26, Lines 603-605: When discussing the superior agility gains in the FCMT group, the manuscript would benefit from comparing its findings with other recent studies that have investigated combined plyometric and change-of-direction training in elite youth athletes. This would provide a richer context for interpreting the results. The authors should consider discussing their findings in light of this study: [Cherni Y, Mzita I, Oranchuk DJ, Dhahbi W, Hammami M, Ceylan HI, Stefanica V, Chelly MS: Effects of loaded vs unloaded plyometric training combined with change-of-direction sprints on neuromuscular performance in elite U-18 female basketball players: a randomized controlled study. Sport Sciences for Health 2025:1-13.]
(2) Page 29, Lines 686-689: The discussion proposes that FCMT's benefits stem from its broad stimulation across the force-velocity curve . This could be expanded by framing the holistic nature of FCMT within a modern paradigm like the 'joint-by-joint' training approach, which emphasizes inter-joint coordination and could also have implications for injury prevention in a multi-directional sport like badminton. Incorporating this perspective is recommended: [Dhahbi W, Materne O, Chamari K: Rethinking knee injury prevention strategies: joint-by-joint training approach paradigm versus traditional focused knee strengthening. Biology of Sport 2025, 42(4):59-65.]
(3) Page 31, Lines 738-744: The limitations section should acknowledge other uncontrolled variables that can significantly influence training adaptation and performance. For instance, the manuscript does not mention monitoring athlete sleep. Given that factors like sleep duration can impact physical and cognitive performance, this should be noted as a potential confounder and an area for future research. It is suggested to add this point and cite the following work: [Bouzouraa E, Dhahbi W, Ferchichi A, Geantă VA, Kunszabo MI, Chtourou H, Souissi N: Single-Night Sleep Extension Enhances Morning Physical and Cognitive Performance Across Time of Day in Physically Active University Students: A Randomized Crossover Study. Life 2025, 15(8):1178.].

**Response:**

(1) We reviewed the discussion section and cited this reference. Since it states that “improvements in athletes' agility may be attributed to enhanced neuromuscular efficiency and more effective utilization of the stretch-shortening cycle,” this mechanism aligns with the findings observed in our study. (Lines 345-347)
(2) Reviewer **#**1 noted in their comments that the section on “broad stimulation effects on the force-velocity curve” lacked direct data support. We conducted extensive literature searches but found no compelling direct data to substantiate this claim. Consequently, we have revised and modified the description of this section. However, upon reviewing the literature cited by Reviewer **#**2, we observed that the biomechanical framework of the ‘joint-by-joint’ training approach mentioned therein shares certain connections with FCMT. The paper states that the core principle of the “joint-by-joint training method” emphasizes inter-joint coordination and optimizes force distribution throughout the entire kinetic chain, highlighting its potential to enhance movement efficiency and reduce the risk of multidirectional movement injuries. This provides deeper theoretical support for FCMT's superiority, and the mentioned improvement in movement efficiency indirectly offers subsequent evidence regarding fatigue reduction. Therefore, when discussing the organizational structural differences between FCMT and CT, we cited this training paradigm as additional supporting evidence. (Lines 306-308)
(3) This is indeed a limitation of this study. We note that participants were required to exert maximum effort during both the training protocols and testing procedures. This necessitates participants maintaining good physical condition and cognitive performance. Although no participants reported significant discomfort and withdrew during testing or training execution, this remains subjective and lacks corresponding physiological evidence. Sleep monitoring is indeed a good idea to address this limitation. We acknowledged the absence of sleep monitoring as a potential confounding factor in the Limitations section and cited this literature. (Lines 394-396)

**We sincerely appreciate the dedicated work of the editors and reviewers, who provided us with a great many valuable suggestions. We hope the revised manuscript meets your expectations and look forward to your positive consideration. If you have any questions, please feel free to contact me at any time.**

**Sincerely,**

Yuhua Gao, PhD

Guangzhou sport University, China

Email: hry13066290079@gmail.com
