# Supplementary material for: Comparative effects of French Contrast Method vs. Complex Training on explosive power and its endurance in youth badminton athletes
Source: PLoS One. 2025 Dec 5;20(12):e0338383. doi: 10.1371/journal.pone.0338383 (PMC12680223; doi:10.1371/journal.pone.0338383)
Supplement: Attachment — Submitted filename: Response_to_Reviewers_auresp_2.docx [file pone.0338383.s003.docx]

#### Response to Reviewers

**Manuscript ID: PONE-D-25-41210**

**Journal: PLOS ONE**

**Title: Comparative Effects of French Contrast Method vs. Complex Training on Explosive Power and its Endurance in Youth Badminton Athletes**

**Data DOI: https://doi.org/10.6084/m9.figshare.30349558.v1**

**Dear Editors of PLOS ONE,**

We extend our gratitude once again to the academic editor and the two reviewers for their valuable feedback. Under your expert guidance, our manuscript has achieved greater balance and scientific rigor. We have revised the manuscript accordingly based on your additional suggestions.

**Below is a detailed point-by-point response.**

**Response to Reviewer #1:**

**Comment 1:**

Abstract:

(1) Line 13 – suggest changing “Compared with the Complex Training methods that have significant and widespread effects on enhancing explosive power…” to “While evidence have indicated complex contrast training can enhance strength and power…”

**Response:**

(1) The changes have been made as suggested. (Lines 13-14)

**Comment 2:**

Introduction:

(1) Line 50 – suggest changing “self-weight” to “bodyweight jumps” and “reduced load” to “assisted jumps”
(2) Line 60 – should be stretch-shortening cycle (not stretch-short).

**Response:**

(1) Yes, the revised content is more appropriate. We have made the necessary changes.(Lines 50-51)
(2) Thank you for your careful review. This was a spelling error, and we have made the necessary correction. (Lines 60)

**Comment 3:**

Methods:

(1) Line 117 – the sessions were separated by closer to 72 hours minimum?
(2) Line 235 – typo of after the Tukey method (..)

**Response:**

(1) After careful inspection and verification, this was found to be a clerical error and has been corrected based on the findings. “closer to 72 hours minimum”. (Lines 117)
(2) Already modified. (lines 235)

**Comment 4:**

Discussion:

(1) Line 328 – I would suggest revising the final two sentences of this paragraph. You didn’t measure fatigue, you measured perceived exertion, which are quite different constructs.
(2) Line 333 – typo here (,,)

**Response:**

(1) Thank you for raising such a detailed question. Indeed, there is a fundamental distinction between “perceived exertion” and “measure fatigue,” and our previous wording placed greater emphasis on “measure fatigue.” However, our revised text highlights “perceived exertion,” making the statement more rigorous. (lines 327-330)
(2) Already modified. (lines 332)

**Comment 5:**

Conclusion:

(1) Line 406 – it would be good to note that it was better over a short time frame, as the study was only 8 weeks (rather than confidently saying it is simply better).
(2) The link you have shared does not work. Please correct to provide raw data.
(3) Table 1 – need to capitalise the words “isometric” and “back”.
(4) Table 3, 4, and 5 – suggest changing “MS” to “1RM Squat”

**Response:**

(1) Indeed, the phrasing “more effective in a shorter period of time” is more precise. We have made the corresponding revisions in the Conclusion and Abstract sections. (lines 28) (lines 407)
(2) We have examined the shared link and confirmed that it was indeed experiencing issues, though the exact cause remains unclear. We have since updated the link and verified that it is now accessible. Here is the updated link: https://doi.org/10.6084/m9.figshare.30349558.v1. (lines 419)
(3) Already modified. (Table 1)
(4) Already modified. (lines 278-279) (Table 3, 4, and 5)

**Response to Reviewer #2:**

**Comment 1:**

Introduction:

(1) Page 18, Line 348: The phrase "strong muscle contractions, as well as effective utilization of the stretch-shorten cycle" is clear. However, the track-changes document indicates the authors intended to remove "centripetal and centrifugal," as agreed upon in their response to Reviewer #1 (Point 7) , but this phrase was not fully removed and remains in the clean version. Please remove it as agreed.

**Response:**

(1) We have reviewed the entire text and found that lines 318-320 appear to contain relevant descriptions. We have removed the expressions in this section.

**Comment 2:**

Methods:

(1) Page 12, Line 221 (and multiple other locations): There is a significant contradiction regarding data availability. The "Response to Reviewers" letter states the data is on Figshare (DOI: 10.6084/m9.figshare.30349558). The "Date Availability Statements" section in the main manuscript (Page 42, Line 988) also lists this Figshare DOI. However, the "Data Availability" section of the submission form (Page 12, Line 221) states: "All the data obtained from the experiments can be retrieved from the ScienceDB database (dataset number: 10.57760/sciencedb. 18250)". Please resolve this contradiction and ensure the correct repository and DOI/accession number are stated consistently throughout all parts of the submission.

**Response:**

(1) We have now ensured that the correct repository and DOI/accession number are consistently stated in all submitted sections. Our links have been updated in the manuscript. Here is the updated link: https://doi.org/10.6084/m9.figshare.30349558.v1.

**Comment 3:**

Discussion:

(1) Page 40, Line 956: The "Practical Applications" section recommends "isometric back squat". This is inconsistent with the term used in the Conclusion. Please ensure consistent terminology.

**Response:**

(1) In the latest version, we have standardized the term “isometric back squat” and ensured consistent terminology.

**Comment 4:**

Conclusion:

(1) Page 16, Line 289 (from Abstract): There is a typographical error: "The findings suggest that The French Contrast..." This should be corrected to "The findings suggest that the French Contrast...".
(2) Page 42, Line 972 / Page 101, Line 2366: The authors correctly adopted the suggestion to change "can" to "may" ("These advantages may be attributed..."). However, the conclusion (Page 40, Line 956) and "Practical Applications" (Page 101, Line 2362) use the term "isometric static deep-back squat." This terminology is not used in the Methods section or Table 1, which use "isometric back squat." Please standardize this terminology throughout the manuscript for clarity.

**Response:**

(1) Thank you very much for your thorough review. This was a printing error, and we have already corrected it. (lines 26)
(2) After our verification, the term “isometric back squat” has been consistently used throughout the entire paper.

**We would like to express our sincere gratitude once again to the editors and reviewers for their diligent work. Your valuable suggestions have significantly enhanced the quality of our manuscript. We hope the revised version meets your expectations and look forward to your favorable consideration. Should you have any questions, please do not hesitate to contact me.**

**Sincerely,**

Yuhua Gao, PhD

Guangzhou sport University, China

Email: hry13066290079@gmail.com
